# Peripheral zone volume ratio (PZ-ratio) is relevant with biopsy results and can increase the accuracy of current diagnostic modality

**DOI:** 10.18632/oncotarget.16753

**Published:** 2017-03-31

**Authors:** Yifan Chang, Rui Chen, Qingsong Yang, Xu Gao, Chuanliang Xu, Jianping Lu, Yinghao Sun

**Affiliations:** ^1^ Department of Urology, Changhai Hospital, Second Military Medical University, Shanghai 200433, China; ^2^ Department of Radiology, Changhai Hospital, Second Military Medical University, Shanghai 200433, China

**Keywords:** biopsy, diagnosis, multiparametric MRI, peripheral zone volume ratio, prostate cancer

## Abstract

The current diagnostic modality of prostate cancer based on prostate specific antigen (PSA) and systematic biopsy is far from ideal in terms of over-diagnosing indolent prostate cancer and missing significant ones. Thus we integrated the peripheral zone volume ratio (PZ-ratio) for diagnostic refinement. This retrospective study included 247 consecutive patients who underwent initial transrectal ultrasound-guided systematic prostate biopsy from April 2014 to November 2015. Prostate volume was determined by semi-automatic contour on axial T2 weighted magnetic resonance imaging (MRI). PZ-ratio was inversely correlated with age (*r* = −0.36, *p* <0.0001). Adding PZ-ratio and MRI findings to the current predictive model (age, PSA density, percent-free PSA) significantly increased diagnostic accuracy in all patients (AUC: 0.871 vs. 0.812, *p* = 0.0059), but not in patient subgroup with PSA 4–10 ng/ml (AUC: 0.863 vs. 0.803, *p* = 0.12). The new model also significantly reduced the number of unnecessary biopsies while missing less significant cancers at a probability threshold of 25%. PZ-ratio is a potential tool in predicting biopsy results, and when added alone or in combination with MRI findings, the diagnostic accuracy can be further enhanced.

## INTRODUCTION

The random nature of transrectal ultrasound (TRUS) guided systematic prostate biopsy sampling and low specificity of prostate specific antigen (PSA) have induced overtreatment of clinically indolent prostate cancer (PCa) and missed diagnosis of significant ones [1]. Therefore, the American Urological Association (AUA) guidelines on PCa recommend against the use of PSA threshold alone as biopsy indicator [2]. Subsequently, PSA density (PSAD), PSA density of the transition zone (PSADTZ) [3] and other modified prostate volume (PV)-related parameters have been proposed and investigated [4–7]. However, the current use of the prostate ellipsoid formula in volume estimation has long been questioned, with a reported 10–20% error compared with prostatectomy specimens [8–10]. Comparatively, PV estimation by contoured MR images is regarded as the most precise noninvasive method available [11].

Although the causal role between benign prostatic hyperplasia (BPH) and PCa is yet to be clarified, enlargement of an aging prostate due to BPH is typically contributed by the transition zone (TZ), while peripheral zone (PZ) is typically considered as age-irrelevant [12]. Because larger glands are related to lower cancer detection rate [13–15], the ratio of zonal volume may have the potential in differentiating PCa patients from BPH individuals. Porcaro et al. [16] reported that the PCa risk in men with TZ/PZ volume ≤ 1 was 2.36 times greater than those > 1. Qi et al. [17] proposed that a cut-off value of 0.47 in TZ index helped to identify unnecessary biopsies. Previous studies using the contoured method on MRI have observed significant correlation between central gland volume fraction and Gleason Score [18], and can better distinguish PCa patients [19]. However, these studies have a relatively small sample size with no further stratification of patients with PSA level of 4–10 ng/ml, and did not perform multivariate correlation analyses. Hence, its efficacy requires further research.

The purpose of the current study was to validate the diagnostic value of PZ-ratio (i.e., PZ volume divided by prostate volume) based upon contoured volume in axial T2 fat saturated MR images, and to evaluate whether incorporating this parameter provides any additional benefit in the current diagnostic modality in detecting clinically significant PCa.

## RESULTS

### Patient characteristics

**Figure 1:**
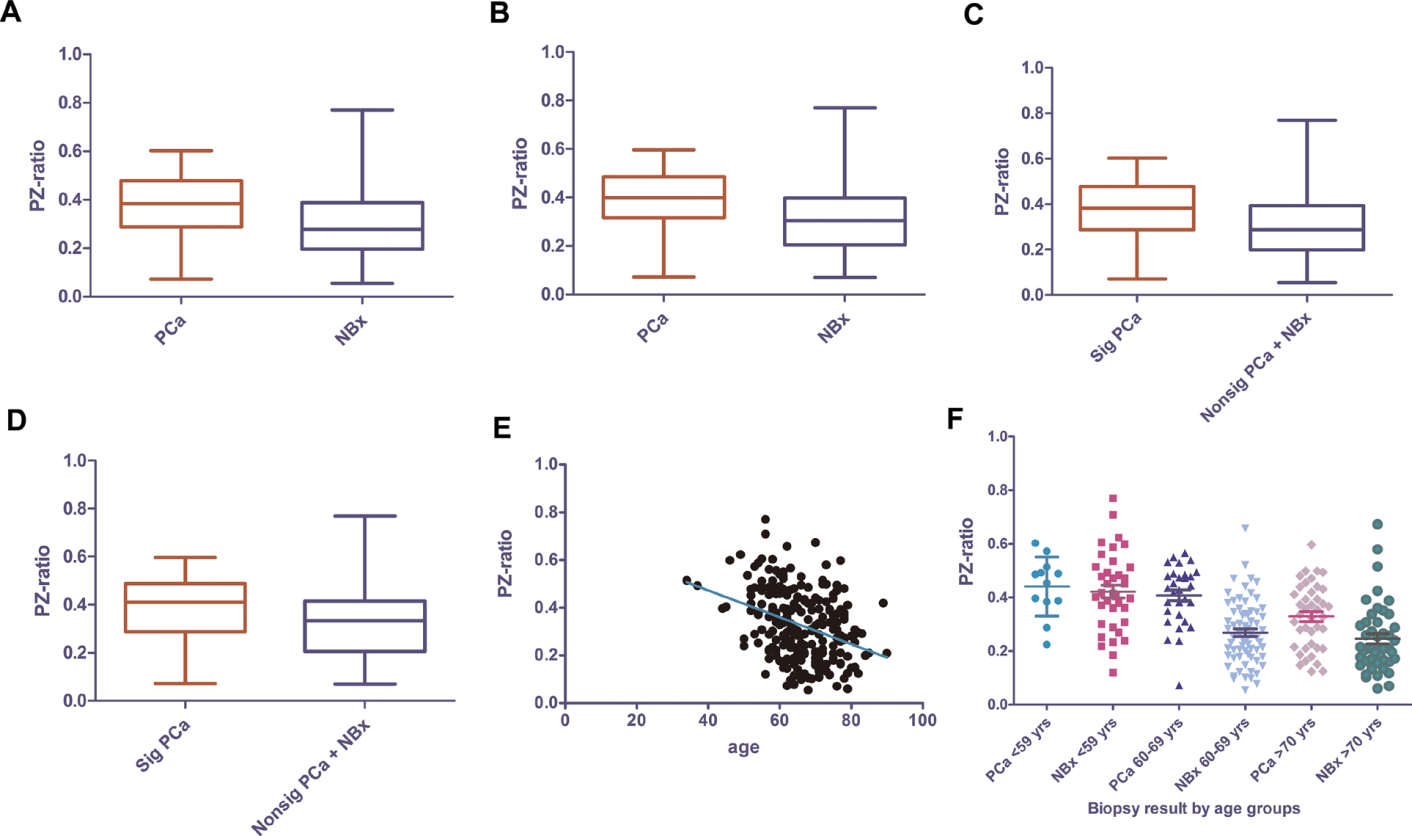
PZ-ratio in PCa and biopsy-negative patients in all PCa of all patient group (**A**) and of patients with PSA of 4–10 ng/ml (**B**)significant PCa vs. insignificant PCa+ negative biopsy of all patient group (**C**) and patients with PSA of 4–10 ng/ml (**D**) and the relevance of PZ-ratio with age in all ages (**E**) and subdivided by age groups (**F**). PZ-ratio, peripheral zone volume ratio; PCa, prostate cancer; NBx, negative biopsy; Sig PCa, significant prostate cancer; nonsig PCa, non-significant prostate cancer.

A total of 247 consecutive patients with PSA ≥ 4.0 ng/ml or PSA < 4.0 ng/ml but with abnormal digital rectal examination (DRE) findings were included in the study, in which 88 cases (35.6%) and 77 cases (31.2%) were diagnosed with PCa and significant PCa, respectively. The median PSA level of PCa patients and biopsy-negative patients were 13.1 (interquartile range (IQR), 8.28 to 21.8) ng/mL and 8.6 (IQR, 6.18 to 13.0) ng/ml, respectively. The median age at biopsy was 68 years in PCa patients and 64 in biopsy-negative patients. The median PZ-ratio was 38.2% (IQR, 28.8% to 47.6%) and 27.7% (IQR, 19.78% to 38.8%) in PCa patients and biopsy-negative patients, respectively (*p* = 0.0001) (Figure 1A). Clinical stage and Gleason Score after biopsy was listed in Table 1. BPH was histologically confirmed in 8 of 88 PCa patients (9.1%) and 89 of 159 biopsy-negative patients (56.0%) (*p* < 0.0001), and 4 of 28 (14.3%) and 38 of 80 (47.5%) in PCa patients and biopsy-negative patients with PSA of 4–10 ng/ml, respectively (*p* < 0.0001).

**Table 1:** Clinical characteristics of men with positive and negative biopsy

	All patients (*n* = 247)			PSA 4.0-10.0 ng/ml (*n* = 108)		
	Positive	Negative	*P* value	Positive	Negative	*P* value
**No. of pts, %**	88 (35.6)	159 (64.4)		28 (25.9)	80 (74.1)	
**Clinically significant Ca**	77 (31.2)	170 (68.8)		19 (17.6)	89 (82.4)	
**Age, median (IQR), years**	68 (61, 76)	64 (59, 71)	P* = 0.0007	66 (61, 77)	62 (57, 68)	P* = 0.0176
**PSA, median (IQR), ng/ml**	13.1 (8.2, 21.8)	8.6 (6.1, 13.0)	P*< 0.0001	7.6 (6.4, 8.5)	7.0 (6.0, 8.5)	P* = 0.3094
**Clinical stage, %**						
** T1c**	10 (11.4)			4 (14.3)		
** T2a/T2b**	53 (60.2)			16 (57.1)		
** T2c**	24 (27.3)			8 (28.6)		
** T3 and above**	1 (1.1)			0 (0.0)		
**Gleason Score, %**						
** < 7**	22 (25.0)			11 (39.3)		
** = 7**	28 (31.8)			9 (32.1)		
** > 7**	27 (30.7)			5 (17.9)		
**Missing data**	11 (12.5)			3 (10.7)		
**Prostate volume, ml**	39.9 (30.7, 56.9)	56.6 (41.0, 75.5)	P* < 0.0001	39.3 (29.6, 53.9)	54.5 (43.2, 69.9)	P* = 0.0007
**%fPSA, %**	11.3 (8.2, 15.7)	15.3 (11.1, 21.5)	P* < 0.0001	11.9 (7.2, 17.4)	15.3 (11.3, 20.8)	P* = 0.0099
**PSAD**	0.32 (0.18, 0.54)	0.15 (0.10, 0.24)	P* < 0.0001	0.18 (0.14, 0.23)	0.14 (0.09, 0.17)	P* = 0.0003
**PZ-ratio, %**	38.2 (28.8, 47.6)	27.7 (19.7, 38.8)	*P*^#^ = 0.0001	39.9 (32.3, 48.3)	30.5 (20.4, 39.5)	*P*^#^ = 0.0196
**Positive MRI, %**	78/88 (88.6)	59/159 (37.1)	P^*^ < 0.0001	24/28 (85.7)	29/80 (36.3)	P^*^ < 0.0001
**BPH rate, %**	8/88 (9.1)	89/159 (56.0)	P^*^ < 0.0001	4/28 (14.3)	38/80 (47.5)	P^*^ < 0.0001

IQR: interquartile range; No. of pts: number of patients; PSA, prostate-specific antigen; %fPSA, percent free PSA; PSAD, prostate-specific antigen density; PZ-ratio: peripheral zone ratio; MRI: magnetic resonance imaging; BPH, benign prostatic hyperplasia. *P* *: Mann-Whitney *U* test; *P*^#^: independent sample test; P^*^: Chi-square test.

### Association between PZ-ratio and other clinical parameters

**Figure 2:**
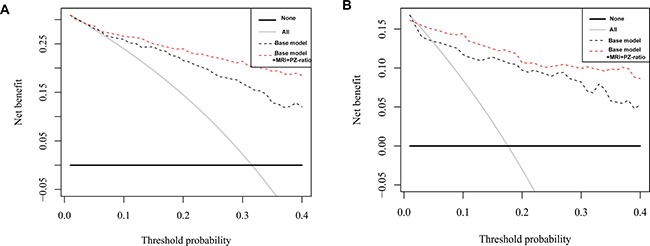
Decision curve analysis of predicting significant PCa with base model vs. base model + MRI + PZ-ratio in all PSA group (**A**) and patients with PSA of 4–10 ng/ml (**B**).

PZ-ratio was correlated with PV (*r* = −0.5698, *p <* 0.0001), percent-free PSA (%fPSA) (*r* = −0.326, *p <* 0.0001) and age (*r* = -0.36, *p <* 0.0001) (Figure 2E) but not with PSA level (*p* = 0.206) or PSAD (*p* = 0.107). Older men were more likely to have lower PZ-ratio (Figure 1E). Since age would significantly influence clinical decision-making, we tested whether the relationship between PZ-ratio and biopsy results varied in patients in different age ranges. PZ-ratio could differentiate PCa from biopsy-negative patients aged 60-69 years and over 69 years (*p <* 0.0001, *p* = 0.0024, respectively) but failed to differentiate prostate cancer from biopsy-negative ones in those younger than 59 years (*p* = 0.691) (Figure 1F).

### Correlation of PZ-ratio and biopsy results

PZ-ratio was effective in predicting PCa in all patients and patients with PSA 4–10 ng/ml. The predicted probability of prostate cancer in all patients (Supplementary Figure 2A) and patients with PSA 4–10 ng/ml (Supplementary Figure 2B) increased with the value of PZ-ratio. The AUC of PZ-ratio was 0.672 (95% CI, 0.603 to 0.741) and 0.659 (95% CI, 0.588 to 0.730) in predicting PCa and significant PCa (Table 2). For patients with PSA of 4–10 ng/ml, the AUC of PZ-ratio was 0.676 (95% CI, 0.557 to 0.795) and 0.642 (95% CI, 0.498 to 0.786) in predicting PCa and significant PCa, respectively. The sensitivity and specificity of PZ-ratio in predicting biopsy result was summarized (Supplementary Table 2). PZ-ratio cutoff value of 34.9% could obtain sensitivity of 67.9% and specificity of 67.5% in predicting significant PCa in patients with PSA of 4–10 ng/ml. To ensure the detection of 90% significant PCa, the PZ-ratio cutoff value should be 20.9%. At a cutoff value of 47.8%, the positive predicted value of PZ-ratio was 0.5. The AUC of PZ-ratio was not higher than traditional predictors, thus we further evaluated if adding PZ-ratio into the diagnostic model would benefit the current clinical practice.

**Table 2:** Diagnostic accuracy of predictors and prediction models in predicting prostate cancer or significant prostate cancer

	Predictors	Predicting PCa		Predicting Significant PCa	
		AUC	95% CI	AUC	95% CI
**All Pts (*n* = 247)**	tPSA	0.679	0.609–0.749	0.722	0.652–0.792
	PZ-ratio	0.672	0.603–0.741	0.659	0.588–0.730
	MRI	0.758	0.707–0.808	0.760	0.711–0.809
	PSAD	0.757	0.694–0.820	0.781	0.717–0.844
	%fPSA	0.657	0.588–0.726	0.653	0.583–0.724
	Model	0.812	0.758–0.866	0.843	0.792–0.893
	Model+PZ-ratio	0.839	0.787–0.891	0.869	0.822–0.917
	Model+MRI	0.854	0.807–0.900	0.874	0.830–0.919
	Model+MRI+PZ-ratio	0.871	0.825–0.917	0.890	0.845–0.934
**Pts PSA 4–10 ng/ml (*n* = 108)**	tPSA	0.565	0.441–0.688	0.522	0.389–0.655
	PZ-ratio	0.676	0.557–0.795	0.642	0.498–0.786
	MRI	0.747	0.663–0.832	0.777	0.704–0.850
	PSAD	0.729	0.620–0.839	0.692	0.555–0.830
	%fPSA	0.664	0.550–0.779	0.632	0.498–0.766
	Model	0.803	0.693–0.913	0.846	0.728–0.964
	Model+PZ-ratio	0.851	0.766–0.936	0.862	0.747–0.976
	Model+MRI	0.822	0.715–0.929	0.888	0.792–0.984
	Model+MRI+PZ-ratio	0.863	0.776–0.951	0.899	0.795–1.000

PCa: prostate cancer; PSA: prostate-specific antigen; PZ-ratio: peripheral zone ratio; MRI: magnetic resonance imaging; OR: odds ratio; CI: confidence interval; AUC: area under the curve.

### Logistic regression analysis

In univariate logistic regression models, PZ-ratio was associated with biopsy result (PCa or non-PCa, and significant PCa or insignificant PCa and benign tissue) in all patients (Supplementary Table 3). While PZ-ratio was only significantly correlated with PCa but only on a borderline significant level with significant PCa (*p* = 0.105), further multivariable logistic regression models including base model (age, PSAD, %fPSA) and base model + PZ-ratio, base model + MRI, base model + PZ-ratio + MRI for PCa and significant PCa, respectively, were listed in Supplementary Table 4.

### Diagnostic accuracy of diagnostic models with PZ-ratio and MRI findings

Adding PZ-ratio into the base model would lead to improvement of AUC in all patients and in patients with PSA 4–10 ng/ml. In the prediction of all PCa in all patients, the AUC of base model, base model + PZ-ratio, base model + MRI, base model + MRI + PZ-ratio was 0.812, 0.839, 0.854, and 0.871, respectively (Table 2, Supplementary Figure 3A). The improvement of AUC was 0.059 (*p* = 0.0059) between base model + MRI + PZ-ratio and base model. Similarly, the AUC of base model + MRI + PZ-ratio was 0.890 in predicting significant PCa, significantly higher than that of the base model (0.890 vs. 0.843, *p* = 0.0227) (Supplementary Figure 3C). However, in patients with PSA 4–10 ng/ml, although the improvement in AUC was similar, the result was not statistically significant in predicting PCa (0.863 vs. 0.803, *p* = 0.125) (Supplementary Figure 3B) or significant PCa (0.899 vs. 0.846, *p* = 0.176) (Supplementary Figure 3D).

### Decision curve analysis for predicting significant PCa

As the decision curve indicated, the base model + MRI + PZ-ratio was superior to the base model in the defined range of interest (15–40% probability) in predicting significant PCa in all patients (Figure 2A) and patients with PSA 4–10 ng/ml (Figure 2B) with a higher net benefit (Supplementary Table 5). Both models outperform the “treat all” strategy of performing biopsies on every patient. Supplementary Table 6 shows the number of significant PCa missed and the reduction in biopsies according to threshold probability for base model and base model + MRI + PZ-ratio. For instance, if a probability threshold of 25% was used in all patients, 3 (3.8%) significant PCa would be missed while avoiding 73 (43.2%) biopsies using the base model; adopting the base model + MRI + PZ-ratio would save 98 (58.1%) of unnecessary biopsies at the cost of missing 4 (5.1%) significant prostate cancers. In patients with PSA 4-10 ng/ml, the base model + MRI + PZ-ratio would save more unnecessary biopsies (64 (71.9%) vs. 59 (66.3%)) while missing less significant cancers (1 (5.3%) vs. 2 (10.5%)) at a probability threshold of 25% for intervention.

## DISCUSSION

Although the etiology of BPH and PCa and their possible correlation is not clearly illustrated [25], epidemiologically, most biopsy candidates are in their 50s to 70s, in which case the incidence of BPH dramatically increases [26]. This contributes, at various extents, to the enlargement of the prostate, and almost exclusively, of TZ [27]. Thus, one may hypothesize that the elevation of PSA in biopsy-negative cases is likely to be contributed by BPH in otherwise healthy males [28]. As a result, PZ-ratio may serve as a volumetric parameter that has the potential to differentiate PCa patients from non-cancer ones, and to provide an additional tool in determining biopsy candidates. In this study, PZ-ratio was able to differentiate PCa patients from BPH men in ages above 60 years, but results were not statistically different in patients under 60 years. Firstly, men aged 50 to 60 may present, if any, only mild BPH. Thus it is reasonable that PZ-ratio in this subgroup may not be markedly different between these two populations. Secondly, the sample size of this younger-aged subgroup may not be sufficient.

When used alone, PZ-ratio was not a good predictor in all patients (AUC = 0.672) and in PSA 4–10 ng/ml subgroup (AUC = 0.676). However, when added to the base model, PZ-ratio alone or in combination with MRI findings were both able to further improve the AUC to up to 0.899. For patients with PSA 4–10 ng/ml, the improvement was not statistically significant. For one, the AUC of all predictors were lower in patients with PSA 4–10 ng/ml than in the overall patients (Table 2); also, because the difference of PZ-ratio between PCa and non-cancer patients in this subgroup was relatively smaller than in the overall population; or, it may result from insufficient sample size; nevertheless, decision curve analysis indicated that the model with PZ-ratio and MRI-findings was able to significantly reduce the number of unnecessary biopsies at the cost of similar or less significant PCa missed in all patients and patients with PSA 4–10 ng/ml.

We believe several aspects of this study strengthened its reliability. Firstly, application of the MRI-based contoured method offers a more precise estimation of prostate volume. Apart from inherent limitations of the ellipsoid formula under TRUS in determining PV, which may underestimate PV by at least 10%, [8] the reproducibility of TRUS is also limited due to inter-observer variability. Comparatively, MRI contour is proven to be more precise (*r* = 0.93 vs. 0.81) than TRUS, [11, 29] and also an easy and simple method with high reproducibility for radiologists and urologists to master after simple training. Our preference of fat saturated T2WI images is explained by the fact that PZ appears hyperintense and more discernable from TZ and other periprostatic tissue [12]. Secondly, it is noted that central zone appears as bilateral basal hypointense zones, or, ‘moustache sign’ on T2 fat saturated images. It is possible that the central zone is mistreated as suspected lesions in the PZ [30], or simply being included as part of PZ, causing additional measurement error. These special attentions may help to achieve more accurate MRI interpretation and more precise measurement results. Thirdly, although this is not the first study focusing on MRI-based PZ-ratio in the diagnosis of PCa, it is, to our knowledge, the first study to test PZ-ratio in a real clinical scenario. It is also the first time that MRI-based PZ-ratio was evaluated in improving current clinical practice and prediction models.

Several limitations of this study should be noted. Firstly, systematic biopsy was performed by three attending urologists in our study, and sampling error may occur. Secondly, the sample size in patients with PSA 4–10 ng/ml was rather inadequate, and further investigations with particular attention to this subgroup is needed. Thirdly, we failed to discriminate TZ PCa from PZ ones. TZ cancers occur far less likely than PZ ones, and are typically not detected by systematic biopsy, because TZ is usually not sampled. Fourthly, since this study was conducted in Chinese population, it concerns us whether the racial difference on PV would influence the efficacy of PZ-ratio. Further multiethnic investigations in western population is needed. Also, the retrospective and single-centered nature of this study has its inherent limitations.

## MATERIALS AND METHODS

### Study population

This study included urology outpatients with PSA levels over 4.0 ng/ml or PSA < 4.0 ng/ml but with suspicious DRE findings. Between April 2014 and December 2015, a total of 247 consecutive patients receiving multiparametric MRI (mpMRI) before 12-core systematic TRUS-guided transrectal biopsy in Changhai Hospital, Shanghai, China, were involved in this study. Ethical approval was acquired from local institutional review board, and informed consent was signed by patients before the study.

### Image technique and biopsy scheme

MR imaging was performed with a 3.0T MR scanner (Magnetom Skyra, Siemens Medical Solutions, Erlangen, Germany). The prostate MRI protocols included T1WI TSE, triplanar (axial, sagittal and coronal) T2WI TSE, diffusion weighted imaging (DWI) and dynamic contrast-enhanced imaging (DCE) by using an 18-channel phased-array coil. Imaging parameters were detailed in Supplementary Table 1. The interpretation of MRI findings was based on PIRADS V2 results performed by an attending radiologist (Q.Y.) with 10 years of experience in prostate MRI. Prostate biopsies were performed by three attending urologists. Additional biopsy cores were taken on suspected spots according to mpMRI findings (i.e., cognitive fusion).

### Pathological review

Two genitourinary pathologists with 21 and 7 years’ experience in prostate histology reviewed all histopathologic slides from prostate biopsy. Clinically significant prostate cancer was defined according to D’Amico's Criteria (i.e., either PSA level >10 ng/ml or Gleason pattern 4 or 5 or clinical stage over T2a) [20].

### Measurement of prostate volume and peripheral zone volume

PV and PZ were semi-automatically segmented on axial fat-saturated T2WI MR images, in OsiriX v5.8.1 (Pixmeo, Geneva, Switzerland), as reported previously [21, 22]. The radiologist (Q.Y.) with 10 years of experience in prostate MRI reviewed and manually corrected the contoured areas, under the supervision of a senior radiologist (J.L.) with over 30 years’ experience on prostate imaging. PV and PZ were calculated by multiplying the sum of contoured area on each slices by slice thickness (Supplementary Figure 1).

### Statistical analysis

Linear correlation analyses were performed to evaluate the association between PZ-ratio and other clinical parameters (age, PSA, %fPSA, PV and PSAD). The predicted probability of biopsy-detected PCa for a given PZ-ratio value was calculated using locally weighted scatterplot (“lowess”) smoothing [23]. Univariate logistic regression analyses were performed to explore the relationship between PZ volume and PZ-ratio with biopsy result (PCa or non-PCa, and significant PCa or insignificant PCa and benign tissue). Multivariate logistic regression analyses were performed to explore if PZ-ratio is an independent predictor of biopsy result. We evaluated the diagnostic accuracy of a base model constructed with clinical predictors (age, PSAD, %fPSA). We also evaluated the diagnostic accuracy of the logistic prediction model with PZ-ratio (base model + PZ-ratio), with MRI results (base model + MRI), and the model with PZ-ratio and MRI results (base model + PZ-ratio + MRI). The predictive accuracy of PZ-ratio, other predictors and prediction models was assessed by area under the curve (AUC) of receiver operating characteristic (ROC) curve. Sensitivity, specificity, positive and negative predicted values were calculated. The comparison of AUC was calculated by *z* test. In addition, decision curve analysis described by Vickers et al. [24] was performed to assess the clinical utility of prediction models with/without PZ-ratio and MRI findings by quantifying the net benefits at a spectrum of threshold probabilities. In this case, we focused on 15–40%, in which clinical decision making is particularly difficult. All *p* values were two-sided and *p <* 0.05 was considered statistically significant. All statistical analyses were performed using MedCalc v.10.4.7.0 (MedCalc Software bvba, Mariakerke, Belgium) and R version 3.1.3 (R foundation for Statistical Computing, Vienna, Austria, http://www.R-project.org) with the Design and Hmisc libraries added.

## CONCLUSIONS

PZ-ratio has the potential as predictor for biopsy results. PZ-ratio is inversely proportionate with age, and can discriminate prostate cancer patients from biopsy-negative ones in men above 60. Alone or in combination with MRI findings, it may offer clinicians additional value based on the current diagnostic modality. Further prospective studies are required to validate its consideration for wide application.

## SUPPLEMENTARY MATERIALS FIGURES AND TABLES


