# Supplementary material for: Peripheral zone volume ratio (PZ-ratio) is relevant with biopsy results and can increase the accuracy of current diagnostic modality
Source: Oncotarget. 2017 Mar 31;8(21):34836–43. doi: 10.18632/oncotarget.16753 (PMC5471015; doi:10.18632/oncotarget.16753)
Supplement: Supplementary file 1 [file oncotarget-08-34836-s001.pdf]

Peripheral zone volume ratio (PZ-ratio) is relevant with biopsy results and can increase the accuracy of current diagnostic modality

Supplementary Materials

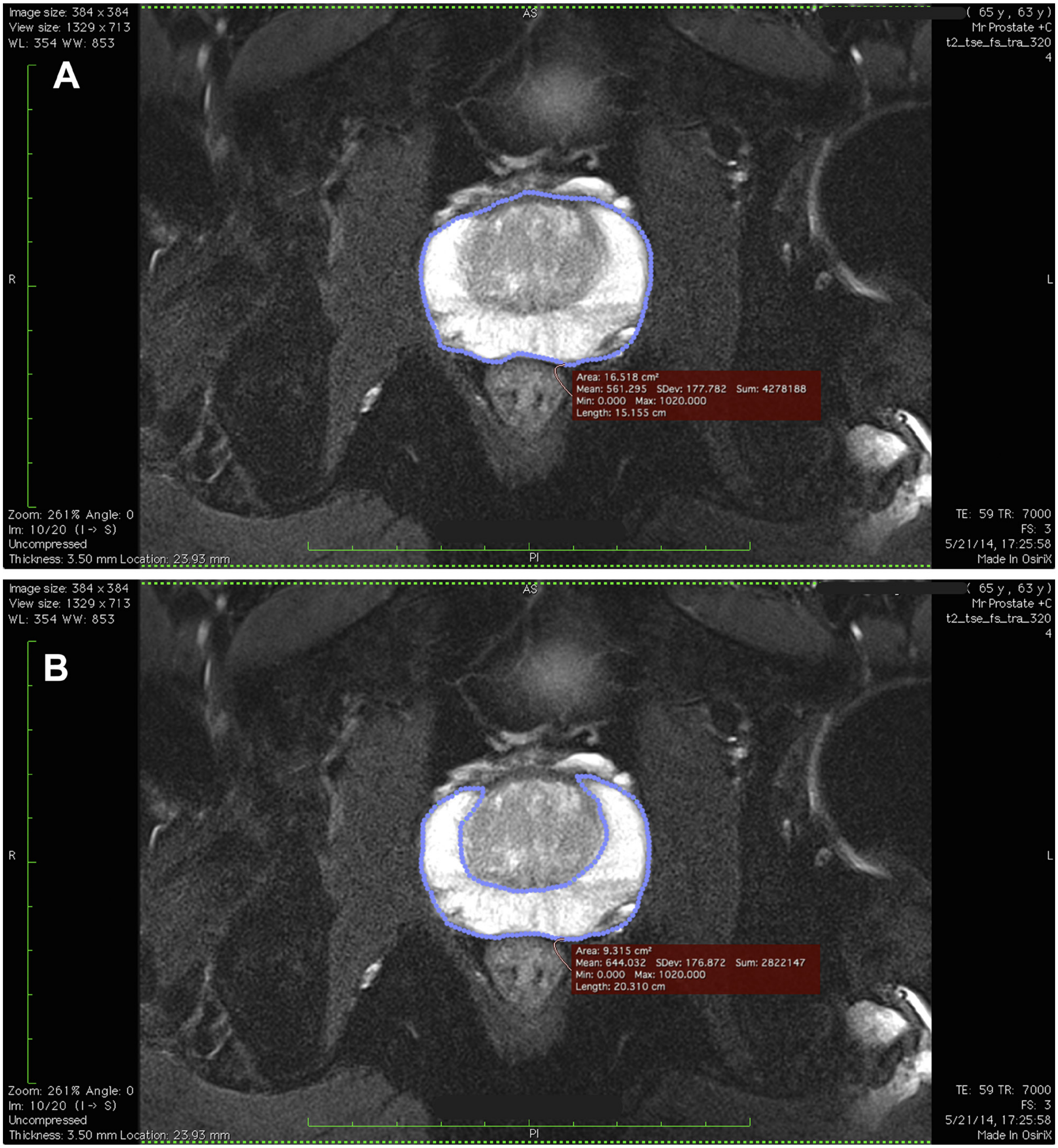

Supplementary Figure 1: Contoured total prostate (**A**) and peripheral zone (**B**) on axial fat-saturated T2WI MR images using a semi-automated segmentation tool.

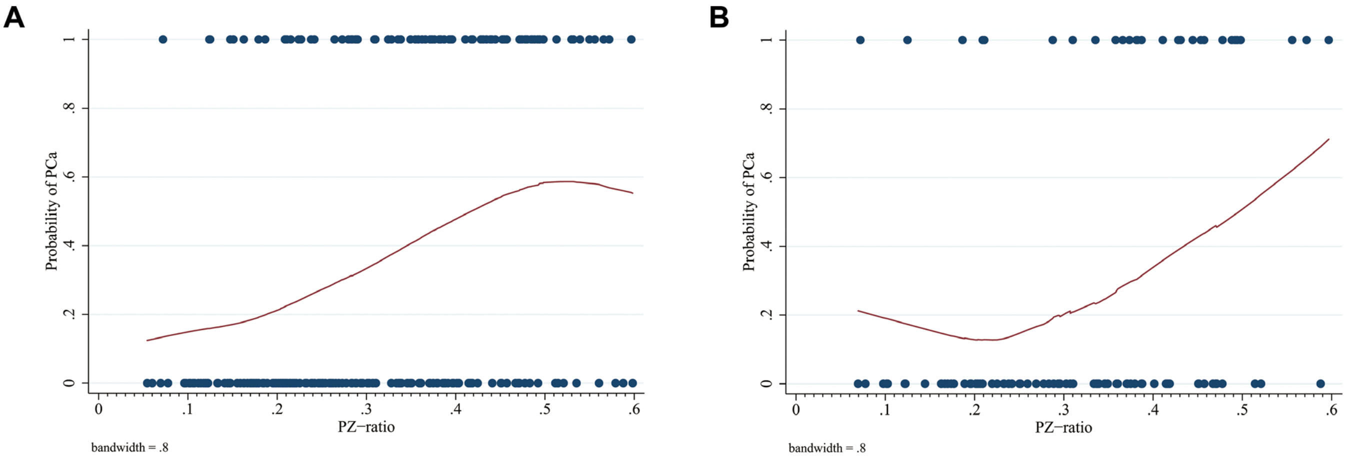

Supplementary Figure 2: PZ-ratio and PCa detection rate in all patient group (**A**) and in patients with PSA of 4–10 ng/ml (**B**).

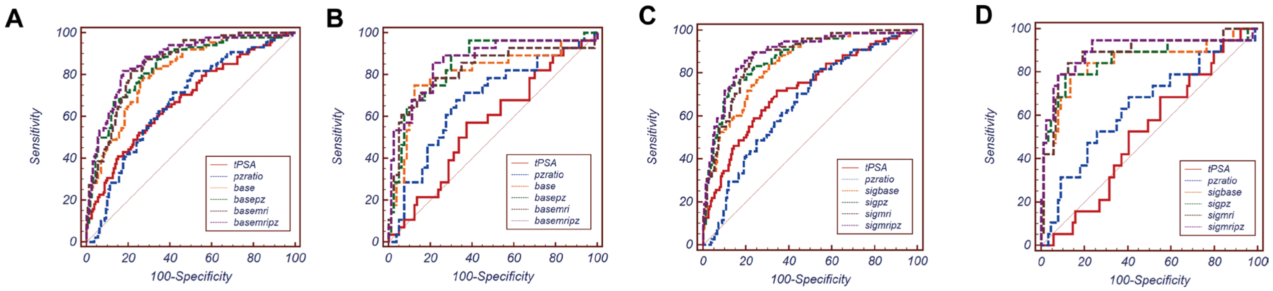

Supplementary Figure 3: ROC curve analysis of PSA, PZ-ratio, base model, base model + PZ, base model + MRI, and base model + MRI + PZ-ratio, in all PCa of all PSA group (**A**), patient with PSA of 4–10 ng/ml (**B**), clinically significant PCa of all PSA group (**C**), and patients with PSA of 4–10 ng/ml (**D**). basepz, base model + PZ-ratio; basemri, base model + MRI; basemripz, base model + MRI + PZ-ratio; sigbase, base model for significant PCa; sigpz, base model + PZ-ratio for significant PCa; sigmri, base model + MRI for significant PCa; sigmripz, base model + MRI + PZ-ratio for significant PCa.

Supplementary Table 1: MRI protocols and main parameters

Protocols	TR/TE (msec)	FOV (mm)	Pixel size (mm^2)	Matrix	Thickness (mm)	TA (sec)
**Axial-T2WI TSE**	4000/104	180	0.5 × 0.5	384 × 384	3.0	129
**Sagittal-T2WI TSE**	7500/97	200	0.8 × 0.8	248 × 256	3.5	178
**Coronal-T2WI TSE**	7500/97	200	0.8 × 0.8	256 × 256	3.5	159
**Axial-T1WI TSE**	750/13	300	0.6 × 0.6	256 × 256	5.0	138
**Axial-DWI SS-EPI**	5100/89	280	1.9 × 1.9	120 × 150	3.0	479
**Axial-DCE FLASH**	3.33/1.23	360	1.9 × 1.9	124 × 192	3.0	313

TR/TE: repetition time/echo time; TA: acquisition time; FOV: field of view; TSE: turbo spin echo; SS-EPI: single shot-echo planar imaging; DWI: diffusion weighted imaging; FLASH: fast low angle shot; *b* values: 0, 50, 100, 150, 200, 500, 800, 1000, 1500, 2000 s/mm^2.

Supplementary Table 2: Sensitivity and specificity of PZ-ratio in predicting prostate cancer or significant prostate cancer in patients with PSA 4–10 ng/ml

	Cutoff	Sensitivity	95% CI	Specificity	95% CI	+LR	−LR	+PV	−PV
**Predicting PCa**	18.7%	89.3	71.8–97.7	17.5	9.9–27.6	1.1	0.6	27.5	82.4
	30.3%	78.6	59.0–91.7	50.0	38.6–61.4	1.6	0.4	35.5	87.0
	36.1%*	67.9	47.6–84.1	67.5	56.1–77.6	2.1	0.5	42.2	85.7
	40.2%	50.0	30.6–69.4	76.3	65.4–85.1	2.1	0.7	42.4	81.3
	47.1%	28.6	13.2–48.7	90.0	81.2–95.6	2.9	0.8	50.0	78.3
**Predicting Significant PCa**	20.9%	89.5	66.9–98.7	27.0	18.1–37.4	1.2	0.4	20.7	92.3
	28.3%	79.0	54.4–93.9	40.5	30.2–51.4	1.3	0.5	22.1	90.0
	34.9%*	68.4	43.4–87.4	59.6	48.6–69.8	1.7	0.5	26.5	89.8
	40.2%	52.6	28.9–75.6	74.2	63.8–82.9	2.0	0.6	30.3	88.0
	47.8%	31.6	12.6–56.6	91.0	83.1–96.0	3.5	0.8	42.9	86.2

PCa: prostate cancer; CI: confidence interval; +LR: positive likelihood ratio; -LR: negative likelihood ratio; +PV: positive predicted value; −PV: negative predicted value.

Supplementary Table 3: Univariate logistic regression models predicting prostate cancer or significant prostate cancer

	All patients	Patients with PSA 4–10 ng/ml
**Predicting Prostate Cancer (OR (95% CI); *p*)**		
**tPSA**	1.0459 (1.0181–1.0746); < 0.001	1.159 (0.876–1.533); 0.302
**PZ-ratio**	47.196 (6.576–338.719); < 0.001	40.750 (1.634–1016.158); 0.024
**MRI**	13.220 (6.353–27.509); < 0.001	10.552 (3.333–33.408); < 0.001
**PSAD**	24.871 (6.338–97.599); < 0.001	71845.571 (95.649–53966146.022); < 0.001
**%fPSA**	0.0004 (0.0001–0.0199); < 0.001	0.0001 (0.000001–0.103); 0.011
**Predicting Significant Prostate Cancer (OR (95% CI); *p*)**		
**tPSA**	1.058 (1.029–1.090); < 0.001	1.058 (0.770–1.453); 0.727
**PZ-ratio**	34.366 (4.679–252.413); < 0.001	18.720 (0.545–643.235); 0.105
**MRI**	15.829 (6.863–36.509); < 0.001	27.771 (3.546–217.488); < 0.001
**PSAD**	37.641 (9.021–157.051); < 0.001	8904.519 (12.544–6320924.785); 0.007
**%fPSA**	0.0004 (0.0001–0.0271); < 0.001	0.0004 (0.00001–1.633); 0.0653

Supplementary Table 4: Multivariate logistic regression models predicting prostate cancer

	All patients		Patients with PSA 4–10 ng/ml	
	**Base Model (OR (95% CI); *p*)**	**Base Model + MRI + PZ-ratio**	**Base Model**	**Base Model + MRI + PZ-ratio**
**Predicting Prostate Cancer (OR (95% CI); *p*)**				
**PZ-ratio**	-	74.954 (4.327–1298.548); 0.003	-	49.196 (0.376–6430.528); 0.117
**MRI findings**	-	7.209 (3.1407–16.5473); < 0.001	-	5.837 (1.614–21.111); 0.007
**Age**	1.086 (1.045–1.128); < 0.001	1.075 (1.028–1.124); 0.002	1.107 (1.040–1.178); 0.001	1.088 (1.012–1.171); 0.023
**PSAD**	13.309 (3.173–55.831); < 0.001	5.669 (1.495–21.489); 0.011	$3.691*10^4$ (12.814–$1.063*10^8$); 0.010	2431.321 (0.499–$1.185*10^7$); 0.072
**%fPSA**	0.001 (0.00005–0.066); 0.002	0.024 (0.0003–1.840); 0.092	0.0002 ($5.2*10^{-8}$–1.066); 0.052	0.013 (0.0001–28.396); 0.354
**Predicting Significant Prostate Cancer (OR (95% CI); p)**				
**PZ-ratio**	-	75.030 (3.652–155.295); 0.005	-	219.123 (0.561–85639.848); 0.046
**MRI findings**	-	7.980 (2.985–21.335); < 0.001	-	10.738 (1.239–93.043); 0.031
**Age**	1.108 (1.063–1.155); < 0.001	1.097 (1.045–1.115); < 0.001	1.178 (1.083–1.281); < 0.001	1.162 (1.057–1.278); 0.002
**PSAD**	25.028 (5.338–117.340); < 0.001	10.538 (2.375–46.758); 0.002	55423.021 (3.933–$7.811*10^8$); 0.025	1395.000 (0.0699–$2.783*10^7$); 0.152
**%fPSA**	0.001 (0.00006–0.114); 0.005	0.032 (0.0003–3.056); 0.139	$2.450*10^{-4}$ ($2.100*10^{-8}$–2.859); 0.082	0.029 ($1.22*10^{-6}$–685.082); 0.491

Supplementary Table 5: Net benefit and reduction in avoidable biopsies in predicting significant prostate cancer for the Model and Model + MRI + PZ-ratio compared to the "treat all" strategy to biopsy every patient for different threshold probabilities in the same range

	Threshold probability (%)		10	15	20	25	30	35	40
**All patients**	Net benefit	Model ∮	25.9	27.7	24.6	20.6	17.9	14.1	12.0
		Model ∮ + MRI + PZ-ratio	26.5	27.9	27.0	25.0	23.7	21.2	19.2
		Treat all	23.9	24.2	19.5	14.2	8.0	1.0	−7.2
	Net reduction in avoidable biopsies	Model ∮	17.4	27.5	28.3	30.8	33.9	33.7	39.1
		Model ∮ + MRI + PZ-ratio	23.1	31.2	38.1	41.4	44.7	45.7	48.8
**Patients with PSA 4-10 ng/ml**	Net benefit	Model ∮	12.0	11.4	9.7	9.0	8.2	5.8	5.2
		Model ∮ + MRI + PZ-ratio	14.0	12.5	10.6	10.2	9.9	9.9	8.6
		Treat all	8.0	3.0	−3.0	−9.9	−17.7	−26.8	−37.3
	Net reduction in avoidable biopsies	Model ∮	29.6	47.2	50.9	56.5	60.5	60.4	63.9
		Model ∮ + MRI + PZ-ratio	53.7	53.4	54.6	60.2	64.5	68.1	69.0

PZ-ratio: peripheral zone ratio. MRI: magnetic resonance imaging; PSA: prostate-specific antigen;
∮ The Model consisted of age, %fPSA and PSAD.

Supplementry Table 6: Number of significant prostate cancer missed and reduction in biopsies according to threshold probability in the range of 10–40% for the base model and base model + MRI + PZ-ratio for all patients and patients with PSA 4–10 ng/ml

	Probability cut-off, %	Model	Significant PCa missed, No. (%)	Unnecessary biopsies** spared, No. (%)
**All patients**	10	Base model ∲	1 (1.3%)	42 (24.8%)
		Base model ∲ + MRI + PZ-ratio	1 (1.3%)	56 (33.2)
	15	Base model ∲	2 (2.6%)	66 (39.0%)
		Base model ∲ + MRI + PZ-ratio	3 (3.8%)	74 (43.8%)
	20	Base model ∲	3 (3.8%)	67 (39.6%)
		Base model ∲ + MRI + PZ-ratio	4 (5.1%)	90 (53.3%)
	25	Base model ∲	3 (3.8%)	73 (43.2%)
		Base model ∲ + MRI + PZ-ratio	4 (5.1%)	98 (58.1%)
	30	Base model ∲	3 (3.8%)	81 (47.8%)
		Base model ∲ + MRI + PZ-ratio	4 (5.1%)	106 (63.0%)
	35	Base model ∲	3 (3.8%)	80 (47.5%)
		Base model ∲ + MRI + PZ-ratio	5 (6.4%)	108 (63.8%)
	40	Base model ∲	5 (6.4%)	92 (54.2%)
		Base model ∲ + MRI + PZ-ratio	6 (7.7%)	115 (67.8%)
**Patients with PSA 4–10 ng/ml**	10	Base model ∲	2 (10.5%)	30 (33.7%)
		Base model ∲ + MRI + PZ-ratio	1 (5.3%)	57 (64.0%)
	15	Base model ∲	2 (10.5%)	49 (55.1%)
		Base model ∲ + MRI + PZ-ratio	1 (5.3%)	57 (63.7%)
	20	Base model ∲	2 (10.5%)	53 (59.6%)
		Base model ∲ + MRI + PZ-ratio	1 (5.3%)	58 (65.2%)
	25	Base model∲	2 (10.5%)	59 (66.3%)
		Base model ∲ + MRI + PZ-ratio	1 (5.3%)	64 (71.9%)
	30	Base model ∲	3 (15.8%)	62 (70.0%)
		Base model ∲ + MRI + PZ-ratio	2 (10.5%)	68 (79.3%)
	35	Base model ∲	3 (15.8%)	62 (70.0%)
		Base model ∲ + MRI + PZ-ratio	3 (15.8%)	71 (79.3%)
	40	Base model ∲	3 (15.8%)	66 (74.1%)
		Base model ∲ + MRI + PZ-ratio	3 (15.8%)	71 (80.3%)

PCa = prostate cancer; PZ-ratio: peripheral zone ratio. MRI: magnetic resonance imaging.
∲The base model consisted of age, %fPSA and PSAD.
** Unnecessary biopsies were defined as: negative biopsy or insignificant prostate cancer.
